# Multiple Pyogenic Liver Abscesses Caused by *Eggerthella lenta* Treated with Ertapenem: A Case Report

**DOI:** 10.1155/2012/718130

**Published:** 2012-03-05

**Authors:** Richard M. Elias, Shiao Yen Khoo, Jakrapun Pupaibool, Ju-Hsien Nienaber, Nathan W. Cummins

**Affiliations:** ^1^Department of Internal Medicine, Mayo Clinic College of Medicine, Rochester, MN 55905, USA; ^2^Division of Infectious Diseases, Department of Medicine, Mayo Clinic College of Medicine, 200 First Street SW, Rochester, MN, 55905, USA

## Abstract

Anaerobic gram-positive bacilli can occasionally be implicated in infections but are difficult to identify in culture by conventional biochemical methods. We report a case of liver abscesses caused by *Eggerthella lenta*, identified via 16S rRNA sequencing in a previously healthy patient, successfully treated with percutaneous drainage and ertapenem.

## 1. Introduction

Anaerobic gram-positive bacilli from the normal human gut flora can occasionally be implicated in invasive diseases. Although *Lactobacillus*, *Clostridium*, and *Propionibacterium* species are more commonly encountered, many other lesser known anaerobic gram-positive bacilli can be involved in clinical infections as well. We report here a case of multiple liver abscesses, in a patient without underlying gastrointestinal disease, caused by *Eggerthella lenta* that was identified via 16S rRNA sequencing, an increasingly used technology that has improved detection of these less commonly seen anaerobic organisms [1].

## 2. Case Report

A 78-year-old retired male mechanical engineer from Arkansas presented with 3-month history of low-grade fever, rigors, and confusion, associated with poor appetite, 15-pound unintentional weight loss, abdominal discomfort, and increased frequency of urination. He denied having nausea, vomiting, or changes in bowel habits. The patient had no significant past medical history, and had no history of sick contacts, recent travel, ingestion of raw food or milk, or known exposures to animals. Urinalysis showed microscopic hematuria, but no pyuria. Although urine culture was negative, he was treated for presumed urinary tract infection with a 7-day course of oral ciprofloxacin without significant improvement, followed by a 14-day course of doxycycline for presumed prostatitis. He was afebrile while taking doxycycline, but fever recurred 3 days after completion of the antibiotic. He was noted by his wife to be confused and found to have blood pressure (90/60 mmHg) on the day of admission.

**Figure 1:**
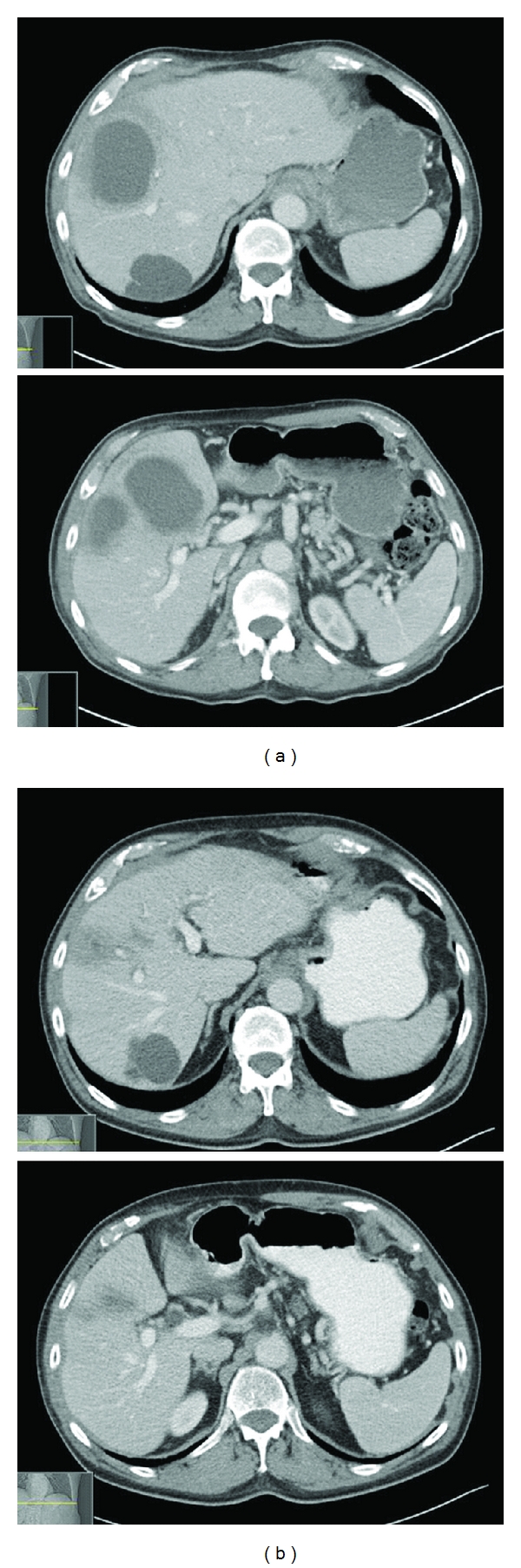
Abdominal CT images showing multiple liver abscesses before treatment (a) and resolution after treatment (b). Of note, patient has a benign liver cyst posteriorly that remained unchanged.

On admission, he had a temperature of 37.3°C, a heart rate of 130 beats/minute, and a blood pressure of 118/72 mmHg. A complete physical examination, including neurological evaluation, was within normal limit. Laboratory results were notable for neutrophilic leukocytosis (white blood cell count of 28.0 × 10^9^/L, absolute neutrophil count of 26 × 10^9^/L), anemia (hemoglobin of 111 g/L), elevated C-reactive protein (31.1 mg/L), and mild hyponatremia (128 mmol/L). His liver and kidney functions were normal, and chest X-ray was unremarkable. Computed tomography (CT) of the head showed mild cerebral degenerative changes. Abdominal CT was significant for three large thick-walled low-density lesions in the right hepatic lobe, with the largest measuring 75 × 55 × 58 mm and multiple smaller low-attenuation lesions within the liver, likely representing multiple hepatic abscesses (Figure 1(a)). Ultrasound-guided aspiration yielded 25 mL of purulent fluid with subsequent placement of two drainage catheters. Gram stain of the purulent fluid showed many white blood cells but no organisms. Fungal and acid-fast smear were negative as well. Intravenous meropenem and vancomycin were empirically begun while awaiting culture results.

Although the patient responded promptly to antimicrobial therapy with resolving confusion, fever, and leukocytosis, his cultures, including blood cultures remained negative. The negative cultures were possibly attributed to the demeclocycline, a tetracycline with some antibacterial properties [3], that the patient received for the treatment of hyponatremia. The patient underwent transesophageal echocardiography to evaluate for endocarditis given concern for septic hepatic emboli from occult source, and it was negative for signs of endocarditis. Due to good clinical response, the patient was discharged on hospital day 7 on ertapenem administered via a peripherally inserted central catheter line, despite negative cultures. Following discharge, the liver aspirate was later reported to be growing a gram-positive bacillus in the anaerobic plate (CDC blood sheep agar) after 6 days of incubation. Identification via PCR-amplified 16S rRNA sequencing technique revealed Eggerthella lenta (Eggerthella lenta strain SECO-Mt75m2 16S ribosome RNA gene sequence: GGATGAACGCTGGCGGCGTGCCTAACACATGCAAGTCGAACGATGAAACCGCCCTCGGGCGGACATGAAGTGGCGAACGGGTGAGTAACACGTGACCAACCTGCCCCCCTCTCCGGGACAACCTTGGGAAACCGAGGCTAATACCGGATACTCCCTCCCCTGCTCCTGCAGGGGTCGGGAAAGCCCAGGCGGAGGGGGATGGGGTCGCGGCCCATTAGGTAGTAGGCGGGGTAACGGCCCACCTAGCCCGCGATGGGTAGCCGGGTTGAGAGACCGACCGGCCACATTGGGACTGAGATACGGCCCAGACTCCTACGGGAGGCAGCAGTGGGGAATTTTGCGCAATGGGGGCAACCCTGACGCAGCAACGCCGCGTGCGGGACGACGGCCTTCGGGTTGTAAACCGCTTTCAGCAGGGAAGAAATTCGACGGTACCTGCAGAAGAAGCTCCGGCTAACTACGT, GenBank # AY937380.1, National Center for Biotechnology Information, U.S. National Library of Medicine). Antimicrobial susceptibility testing was not performed because of inadequate growth. At followup, the patient was doing well clinically with radiographic resolution of the hepatic abscesses after a 6-week course of ertapenem (Figure 1(b)). Colonoscopy after resolution of infection showed extensive diverticulosis distal to the hepatic flexure.

## 3. Discussion

**Figure 2:**
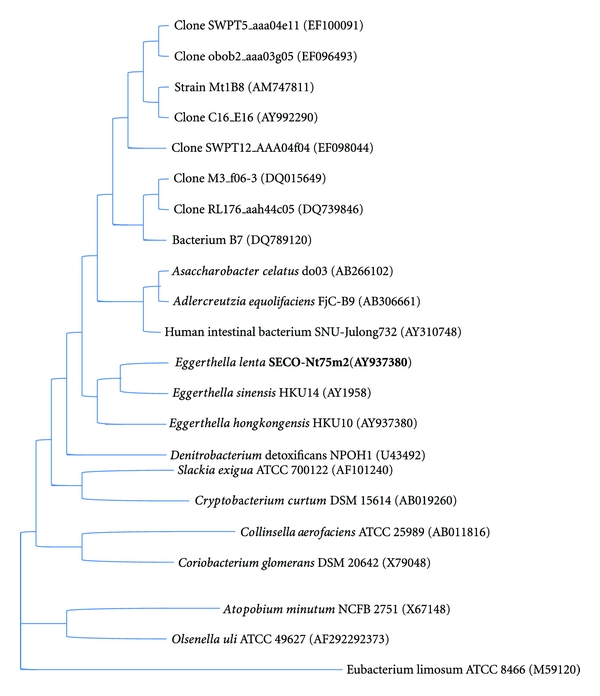
Phylogenetic position of strain *Eggerthella lenta* SECO-Mt75m2 among members of the family Coriobacteriaceae based on 16S RNA sequencing. Adapted with permission from Clavel et al. [2].

We report a rare case of liver abscesses caused by *Eggerthella lenta* in a patient without a history of gastrointestinal or hepatobiliary disease that responded to treatment with ertapenem. *Eggerthella lenta* is a member of the family Coriobacteriaceae (Figure 2) [2]. It is part of the commensal flora of the gastrointestinal tract and the environment such as soil and water [4]. Previously known as *Eubacterium lentum* and first isolated from a rectal tumor by André Prévot in 1938, the bacteria was given its own genus *Eggerthella* in 1999 based on 16S rRNA gene sequencing [5]. It is a nonsporulating, obligate anaerobic gram-positive bacillus that occurs singly, in pairs, and in short chains [4]. Biochemically, *E. lenta* is catalase- and indole negative and does not hydrolyze gelatin but reduces nitrate. The products of glucose fermentation (acetate, lactate, and succinate) differentiate it from other phenotypically similar species such as *Propionibacterium* (propionate), *Lactobacillus* (lactate), *Bifidobacterium* (acetate and lactate), and *Actinomyces* (succinate) [6].

Clinically,* E. lenta* is typically associated with gastrointestinal diseases, including malignancies and hepatobiliary diseases. It has also been implicated in genital tract infections such as pelvic inflammatory disease [6, 7]. It has been isolated from appendices (both inflamed and noninflamed), intra-abdominal abscesses, intestinal tumors, peritoneal fluid, the female genital tract, sinusitis, decubitus ulcers, oropharyngeal abscesses, and even the walls of an abdominal aortic aneurysm [8]. Lau et al. reported 5 cases of *E. lenta *bacteremia, the sources of which were attributed to gastrointestinal infection, pelvic inflammatory disease, and infected decubitus ulcers [9]. Interestingly, the initial source of infection for our patient is unknown, as he had no obvious underlying gastrointestinal or hepatobiliary disease. A possibility is the extensive diverticulosis noted on colonoscopy that could have led to transient polymicrobial bacteremia with resultant seeding of the liver. But cultures only grew *E. lenta* due possibly to receipt of antibiotics without anaerobic activity prior to presentation.

Although *Eggerthella* bacteremia can lead to high morbidity and mortality including septic shock, multiorgan failure, and death in 20–40% of the cases [7], the natural history and prognosis of *E. lenta* clinical infections aside from bacteremia has not been well described. Intra-abdominal abscesses often require percutaneous or surgical drainage [7], as in our patient. Due to potential for high mortality, prompt identification of the microorganism and source and initiation of appropriate antibiotics are essential.

However, as with other medically important gram-positive, non-spore-forming anaerobic bacilli, *E. lenta* is difficult to culture and identify in microbiology laboratories for many reasons. They are fastidious, slow growing, and phenotypically labor intensive to speciate [9]; sometimes it may take 2 to 6 weeks for identification [1]. For our patient, the gram stain of purulent liver abscess aspirate was negative and required 6 days to return a positive culture and 2 more days for further identification. Most often cultured from polymicrobial sources such as the gastrointestinal and genital tracts, *E. lenta* is also frequently overgrown by less fastidious, aerobic organisms [4]. The paucity of cases of *E. lenta* infections likely reflects the above difficulties, in addition to the postulated low pathogenicity of the organism [8].

Sequencing of 16S rRNA has become an increasingly important and widely used tool for rapid and accurate identification of bacterial isolates. Because the 16S RNA gene is highly conserved within species and amongst members of a particular genus, this sequencing technology has been instrumental in reclassification and identification of novel genera and species [1]. RNA sequencing can be labor intensive and hindered by the quality of the reference sequence database and the complexity in interpreting the sequence phylogeny. However, with the availability of PCR, automated RNA sequencing technology, as well as Clinical and Laboratory Standards Institute (CLSI) DNA target sequencing guidelines [10], the 16S rRNA sequencing has become a more reliable and widely used identification method for difficult microorganisms in a matter of 1-2 days [1]. The improved diagnostic efficiency facilitates prompt initiation of appropriate and life-saving antibiotics.

Antibiotic susceptibilities were not performed on the isolate reported herein. However, our patient demonstrated clinical improvement following initial empiric treatment with meropenem and vancomycin, followed by ertapenem monotherapy. Few studies have evaluated the susceptibilities of *E. lenta *[4, 8, 11]. Based on agar dilution and E-test methods susceptibility studies, in general, *E. lenta *are susceptible to amoxicillin-clavulanic acid (MIC ≤ 2/1), metronidazole (MIC ≤ 2), and clindamycin (MIC ≤ 0.5) [4, 8, 11], according to Clinical and Laboratory Standards Institute (CLSI) MIC breakpoints for anaerobes. With its known anaerobic activity, moxifloxacin also demonstrated good activity against *E. lenta *(MIC ≤ 1) [11]. Although ertapenem susceptibilities were not found in the literature, imipenem demonstrated good activity (MIC ≤ 0.5) [11]. Surprisingly, the MIC for penicillin (≤1) is in the intermediate breakpoint, and one recent report noted resistance to piperacillin-tazobactam (MIC = 32) [4]; hence clinicians should refrain from the use of penicillin. *E. lenta *also appears to be uniformly resistant to cefotaxime (MIC > 256) [4]. Overall, the evidence suggests that amoxicillin-clavulanic acid, metronidazole, clindamycin, moxifloxacin, and carbapenems appear to have good activity against *E. lenta.*

This case illustrates that *E. lenta *can cause liver abscesses in the absence of obvious risk factors, despite the postulated low virulence of anaerobic gram-positive bacilli, and that it responds to treatment by ertapenem. Typically time consuming to identify by traditional phenotypic techniques, anaerobic gram-positive bacilli can now be more quickly identified by16S rRNA gene sequencing, helping to increase our understanding of the role of these bacteria in clinically significant infections.
